# Genotypic and Phenotypic Characterization of Chikungunya Virus of Different Genotypes from Malaysia

**DOI:** 10.1371/journal.pone.0050476

**Published:** 2012-11-27

**Authors:** I-Ching Sam, Shih-Keng Loong, Jasmine Chandramathi Michael, Chong-Long Chua, Wan Yusoff Wan Sulaiman, Indra Vythilingam, Shie-Yien Chan, Chun-Wei Chiam, Yze-Shiuan Yeong, Sazaly AbuBakar, Yoke-Fun Chan

**Affiliations:** 1 Tropical Infectious Diseases Research and Education Centre, Department of Medical Microbiology, Faculty of Medicine, University Malaya, Kuala Lumpur, Malaysia; 2 Department of Parasitology, Faculty of Medicine, University Malaya, Kuala Lumpur, Malaysia; Agency for Science, Technology and Research - Singapore Immunology Network, Singapore

## Abstract

**Background:**

Mosquito-borne Chikungunya virus (CHIKV) has recently re-emerged globally. The epidemic East/Central/South African (ECSA) strains have spread for the first time to Asia, which previously only had endemic Asian strains. In Malaysia, the ECSA strain caused an extensive nationwide outbreak in 2008, while the Asian strains only caused limited outbreaks prior to this. To gain insight into these observed epidemiological differences, we compared genotypic and phenotypic characteristics of CHIKV of Asian and ECSA genotypes isolated in Malaysia.

**Methods and Findings:**

CHIKV of Asian and ECSA genotypes were isolated from patients during outbreaks in Bagan Panchor in 2006, and Johor in 2008. Sequencing of the CHIKV strains revealed 96.8% amino acid similarity, including an unusual 7 residue deletion in the nsP3 protein of the Asian strain. CHIKV replication in cells and *Aedes* mosquitoes was measured by virus titration. There were no differences in mammalian cell lines. The ECSA strain reached significantly higher titres in *Ae. albopictus* cells (C6/36). Both CHIKV strains infected *Ae. albopictus* mosquitoes at a higher rate than *Ae. aegypti*, but when compared to each other, the ECSA strain had much higher midgut infection and replication, and salivary gland dissemination, while the Asian strain infected *Ae. aegypti* at higher rates.

**Conclusions:**

The greater ability of the ECSA strain to replicate in *Ae. albopictus* may explain why it spread far more quickly and extensively in humans in Malaysia than the Asian strain ever did, particularly in rural areas where *Ae. albopictus* predominates. Intergenotypic genetic differences were found at E1, E2, and nsP3 sites previously reported to be determinants of host adaptability in alphaviruses. Transmission of CHIKV in humans is influenced by virus strain and vector species, which has implications for regions with more than one circulating CHIKV genotype and *Aedes* species.

## Introduction

Chikungunya virus (CHIKV) is an alphavirus from the *Togaviridae* family, which is transmitted by both *Aedes aegypti* and *Ae. albopictus*. It is a single-stranded, positive sense RNA virus, with a genome of about 11.8 kb, and two open reading frames encoding the nonstructural (nsP1-nsP2-nsP3-nsP4) and structural polyproteins (C-E3-E2-6K-E1). CHIKV causes fever, rash, and arthralgia, with the latter sometimes lasting for months. Phylogenetic analysis shows that there are three major CHIKV genotypes: West African, East/Central/South African (ECSA), and Asian [1]. After its identification in Tanzania in 1952 [2], CHIKV caused sporadic outbreaks in Asia and Africa, punctuated by years of apparent inactivity [1]. During interepidemic periods, CHIKV may be maintained in a sylvatic cycle in non-human primates [3], [4]. However, since 2005, ECSA strains from East Africa have spread to the Indian Ocean [5] and India [6], and then onwards to Europe [7], Asia [8]–[11], and North America [12], affecting millions. Adaptation of the virus to the secondary vector *Ae. albopictus* contributed to this unprecedented spread [13].

Malaysia is located in Southeast Asia, which is endemic for CHIKV. Although low levels of seroprevalence were noted in human populations as early as the 1960s [14], CHIKV was only identified for the first time during an outbreak in Klang in 1998 [15]. A further outbreak occurred in Bagan Panchor, a fishing village in Perak state, in 2006 [16], [17]. The causative CHIKV strains were of the Asian genotype, as were strains isolated from wild macaques in Malaysia in 2007 [4], suggesting that this genotype is endemic in Malaysia. A third outbreak in Ipoh in 2006 was the first to be caused by the ECSA genotype [18]. These three outbreaks were each limited to single sites, affecting about 300 people in total. CHIKV of the ECSA genotype then caused Malaysia’s first nationwide outbreak in 2008–2010, affecting over 10,000 people [11].

Malaysia therefore has two CHIKV genotypes: the previously isolated Asian and the recently imported epidemic ECSA genotypes, which have clear epidemiological differences. The Asian genotype caused restricted outbreaks with no reported severe disease, while the ECSA genotype caused an epidemic extending throughout the country. Comparative laboratory data between CHIKV genotypes is limited, but may have important implications for disease occurrence in countries with more than one circulating genotype. To gain insight into the observed epidemiological differences between the two CHIKV genotypes found in Malaysia, we studied their genotypic and phenotypic differences in cell lines, *Ae. albopictus*, and *Ae. aegypti* mosquitoes.

**Table 1:** Amino acid differences between Malaysian Chikungunya strains of Asian and East/Central/South African genotypes.

Protein	Length (amino acids)	Number of amino acid differences (%)	Amino acid differences
nsP1	535	13 (2.4)	S3P, S34P, T128K, V153I, M253K, T376M, G454S, R473S, A478T, N486D, Q488R, Q491R, H507R
nsP2	798	12 (1.5)	L16P, S54N, S218T, L273Q, M338K, H374Y, V466M, V486I, L539S, I756V, S768N, A793V
nsP3	530	35 (6.6)	T77S, G117R, V175I, I176V, V213M, N283S, V303T, V331A, R332Q, V334A, M336T, T337I, A349V, T353I, del376-382THTLPST, I383T, I413T, Q434L, A437V, I449M, R452Q, I457T, T458A, V459T, L461P, S462N, P471S, D483N, D484E
nsP4	611	13 (2.1)	L42A, T58M, T75A, K85R, A90S, V101T, R235Q, T254A, R271K, D280E, A366T, Q500L, A582V
non-structural polyprotein	2474	73 (3.0)	
C	261	8 (3.1)	P23S, V27I, K37Q, V48A, K73R, R78Q, M81T, V93A
E3	64	4 (6.3)	K33E, S44R, R60H, R62Q
E2	423	18 (4.3)	I2T, H5N, G118S, R149K, A157V, A164T, S194G, D205G, S207N, S248L, K252Q, V/L255I, T312M, I317V, R318V, S375T, V384M, V386A
6K	61	4 (6.5)	V8I, M45T, T47A, L52M
E1	440	10 (2.3)	S72N, T98A, A145T, E211K, S225A, A226V, M269V, D284E, S304P, P397L
structural polyprotein	1249	44 (3.5)	

Amino acid differences are reported following alignment of sequences obtained in this study from the Bagan Panchor strains MY/06/37348 and MY/06/37350 (Asian genotype), and the Johor strains MY/08/065 and MY/08/068 (ECSA genotype). The first amino acid named is found in the Asian strains, while the second amino acid is found in the ECSA strains. Differences at sites reported to be mosquito adaptation determinants in other alphaviruses are underlined.

**Figure 1:**
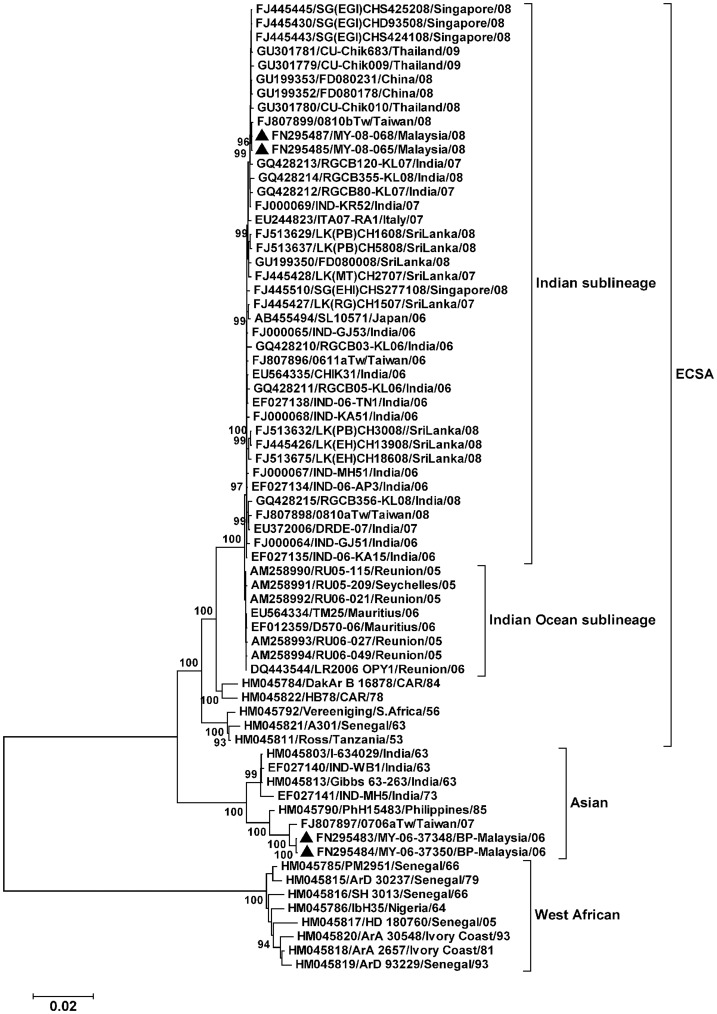
Phylogenetic analysis of Chikungunya virus. Full coding sequences of CHIKV were used. The maximum likelihood tree was constructed using the general time reversible model with proportion of invariant sites, and inferred following bootstrap analyses using 1000 replicates. Branch lengths are measured in the number of substitutions per site, as shown in the scale. Strain names are in the format: accession number/strain name/country of isolation/year of isolation. The Malaysian isolates sequenced in this study are indicated by (▴). CHIKV has three main genotypes: West African, Asian, and East/Central/South African (ECSA); the latter has at least two sublineages, Indian Ocean and Indian.

## Materials and Methods

### Ethics Statement

One of the authors (WYWS) consented to donate blood to feed the mosquitoes.

### Virus Isolates

The CHIKV isolates sequenced in the study were two isolates from the Bagan Panchor outbreak in 2006 (MY/06/37348 and MY/06/37350, Asian genotype), and two isolates from Johor during the nationwide outbreak of the ECSA genotype in 2008 (MY/08/065 and MY/08/068). Isolates MY/06/37348 and MY/08/065 were used for study of replication in cells and mosquitoes. The Bagan Panchor and Johor isolates were isolated from patient serum, and passed not more than three times in Vero cells (African green monkey kidney, ATCC CCL-81). Virus stocks were prepared by freeze-thawing the infected cells once, centrifuging the suspension at 40,000 g and storing the filtered supernatants at −80°C.

### Sequencing

RNA was extracted from 140 µl cell culture supernatant using QIAamp Viral RNA Mini Kit (Qiagen, Germany). Isolates MY/06/37348, MY/06/37350, MY/08/065 and MY/08/068 were selected for sequencing of the full genome coding regions. The sequences have been deposited with the accession numbers FN295483, FN295484, FN295485, and FN295487, respectively. Amplification was performed with Access RT-PCR system (Promega, USA) using previously published primers, with modifications (Table S1). The amplified DNA fragments were purified with QIAquick PCR Purification kit (Qiagen), and sequenced using BigDye Terminator Cycle Sequencing kit (Applied Biosystems, USA) with a 3730XL Genetic Analyzer (Applied Biosystems, USA).

### Phylogenetic Analysis

Consensus full coding sequences were assembled using Geneious 5.1 (Biomatters Ltd, New Zealand), and aligned with other CHIKV genomes available from GenBank. Using jModeltest 0.1.1 [19], the best-fitting substitution model was found to be the general time reversible model with proportion of invariant sites (GTR+I). The maximum likelihood tree was drawn using MEGA5 [20]. The strength of the phylogenetic tree was estimated by bootstrap analyses using 1000 random samplings.

### Replication Kinetics of Viruses in Cells

The mammalian cell lines Vero and RD (human rhabdomyosarcoma, ATCC CCL-136), and the mosquito cell lines C6/36 (*Ae. albopictus*, ATCC CCL-1660) and CCL-125 (*Ae. aegypti*, ATCC CRL-125) were used to compare the replication of the isolates MY/06/37348 and MY/08/065. These cells were selected to represent the hosts of CHIKV. The same batch of each cell line was used for each comparative experiment. Vero and C6/36 cells were maintained in EMEM supplemented with 10% FBS, 2 mM L-glutamine, 1X non-essential amino acids, 100 units/ml penicillin and 100 µg/ml streptomycin. CCL-125 cells were maintained in EMEM supplemented with 20% FBS, 1 mM sodium pyruvate, 2 mM L-glutamine, 1X non-essential amino acids, 100 units/ml penicillin and 100 µg/ml streptomycin. RD cells were maintained in DMEM supplemented with 10% FBS, 2 mM L-glutamine, 1X non-essential amino acids, 100 units/ml penicillin and 100 µg/ml streptomycin. Mammalian and mosquito cells were incubated at 37°C and 28°C respectively, in the presence of 5% CO_2_. Cells were seeded in 24-well plates at a density of 5×10^4^ cells/well (Vero and RD) or 1×10^5^ cells/well (C6/36 and CCL-125), with 500 µl media. After overnight incubation, cells were infected with CHIKV at MOI of 0.1, and rocked at room temperature for 1 hour. Virus titration performed at this time-point was considered to be at 0 hours post-infection (hpi). CCL-125 cells could not be successfully infected with an MOI of 0.1, and were infected at MOI of 1. Virus inoculums were then removed, cells rinsed twice with serum-free medium, and medium supplemented with 2% FBS was added. Supernatant samples were collected at 8-hourly time-points until 72 hours, and at 96 hours, and stored at −80°C for later titration. At least 3 independent experiments were performed.

**Figure 2:**
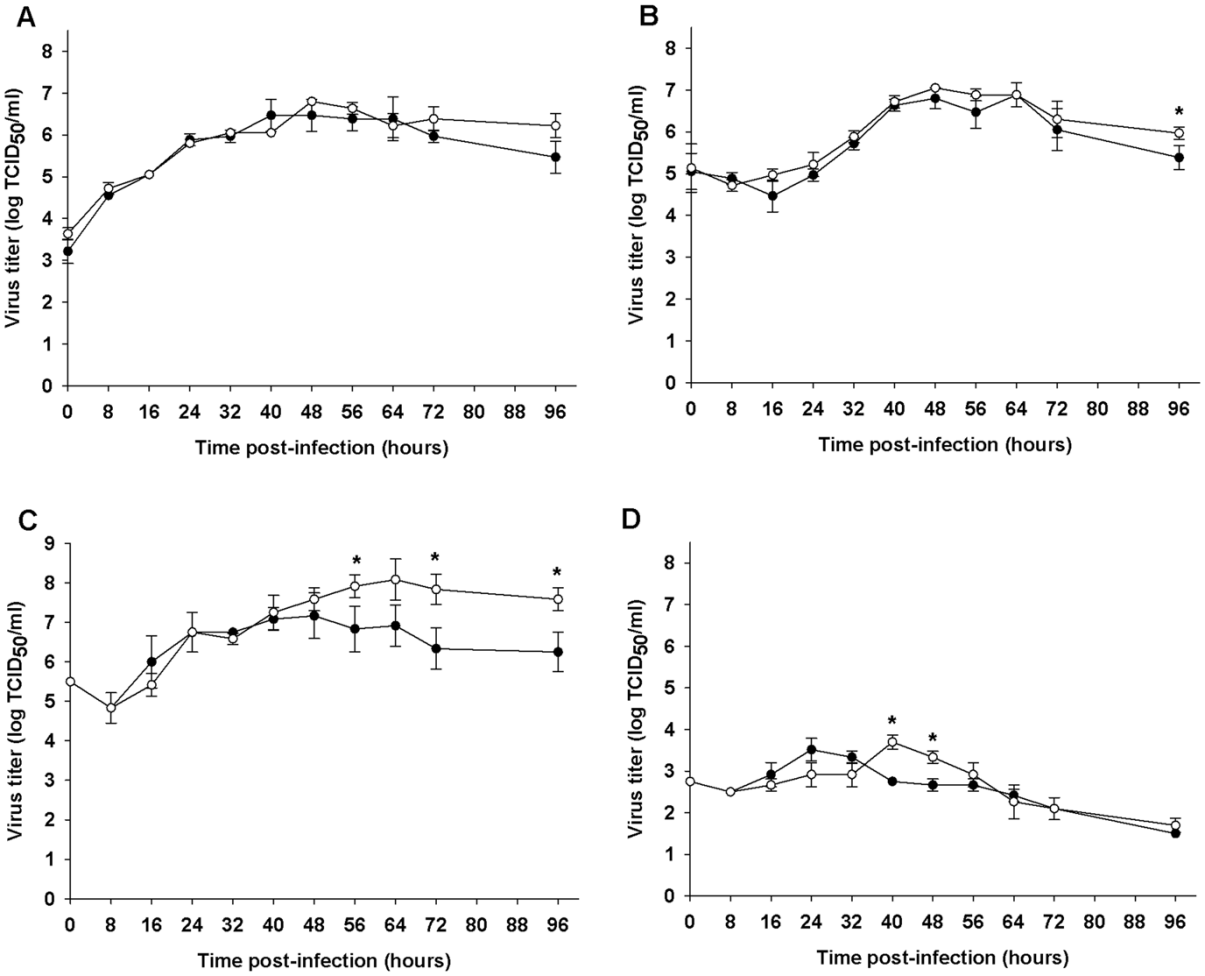
Comparative replication kinetics of Malaysian CHIKV strains in cells. The CHIKV isolates MY/06/37348 (Asian, •) and MY/08/065 (ECSA, ○) were used. Replication was measured by virus titration using a TCID_50_ assay in (A) Vero, (B) rhabdomyosarcoma, (C) C6/36, and (D) CCL-125 cells. Means ± SD of 3 independent experiments are plotted. Asterisks indicate significant differences (*p*<0.05) at the same time-points.

### Replication Kinetics of Viruses in *Ae. aegypti* and *Ae. albopictus*

*Ae. albopictus* (Bangsar strain, collected in Kuala Lumpur) and *Ae. aegypti* (University Malaya strain, collected in Kuala Lumpur) mosquitoes, established in the Department of Parasitology, University of Malaya, were used in this study. Each mosquito species was fed blood meals containing either MY/06/37348 or MY/08/065. Blood was donated by one of the authors (WYWS) and shown to be CHIKV PCR-negative and neutralisation assay-negative. Virus strain MY/06/37348 or MY/08/065 at 5.5 log_10_ TCID_50_/ml was diluted 1∶10 in the blood. Mosquitoes aged 3–6 days were starved overnight before being exposed to the blood meals using gerbil skin attached to a glass feeder. Blood meals were maintained at 37°C throughout the 1.5 hr feeding period. After feeding, fully engorged mosquitoes were sorted on ice into polystyrene cups for each subsequent planned time-point (days 0, 1, 2, 3, 5, 7, and 10). For the negative controls, 3 mosquitoes fed with clean blood were kept aside for sampling on each of days 0, 2 and 5. All mock-infected and infected mosquitoes were fed with 10% sucrose supplemented with vitamin B complex, and kept at 28±1°C with 80% relative humidity and a 12 hr:12 hr photoperiod. Each experiment with a different virus-mosquito combination was carried out separately.

At each time-point, after discarding dead mosquitoes, mosquitoes were killed by freezing, and dissected to remove midguts and salivary glands separately. A clean pair of needles, after being soaked in 70% alcohol, was used for dissecting each mosquito. Organs were placed in individual tubes with 1.4 mm Ceramic Beads (OMNI International, USA) and 0.5 mL of serum-free medium, and homogenised at 5000 rpm for 10 seconds with 1 cycle using a Precellys 24 homogeniser (Bertin Technologies, France). For day 0 of the infected mosquitoes, and days 0, 2 and 5 for mock-infected mosquitoes, 3–5 whole mosquitoes were suspended in 1 mL serum-free medium, before homogenisation. Virus titration was performed for each homogenate in triplicate.

For each experimental time-point, the infection rate was defined as the number of midguts with detectable virus titre divided by the number of mosquitoes sampled. The dissemination rate was defined as the number of salivary glands with detectable virus titre divided by the number of midguts with detectable virus titre.

### Virus Titrations

The virus titres from cell culture supernatants and mosquito homogenates were quantified. Serial 10-fold dilutions were made of samples collected at each time-point, and 100 µl samples of each dilution were added to triplicate wells of 96-well plates containing 80–90% confluent monolayers of Vero cells. Viral titres were determined as TCID_50_/ml using the Reed-Muench method after incubation at 37°C for 7 days.

### Statistical Analysis

Virus titres were log-transformed before Student’s *t*-test was used to compare means. Mosquito infection and dissemination rates were compared with Fisher’s exact test. All statistical analyses were performed using GraphPad Prism 5 (GraphPad Software, USA).

**Figure 3:**
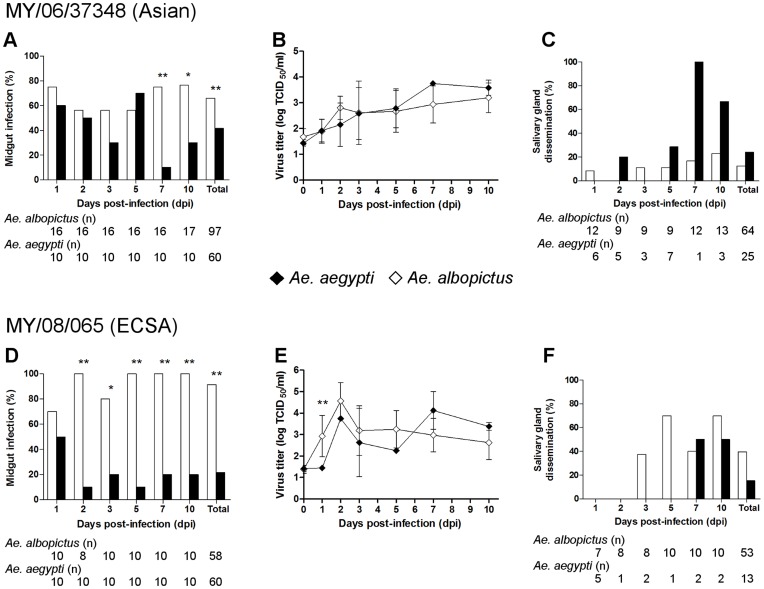
Comparative infection, replication kinetics, and dissemination of each Malaysian CHIKV strain in *Ae. aegypti* (⧫, black bars) or *Ae. albopictus* (⋄, white bars). The CHIKV isolates MY/06/37348 (Asian) and MY/08/065 (ECSA) were used. Following ingestion with MY/06/37348, infection rates of midguts of mosquitoes (A), replication in midguts as measured by a TCID_50_ titration assay with plotted means ± SD of triplicates (B), and dissemination rates in salivary glands of mosquitoes (C) were determined. Following ingestion with MY/08/065, midgut infection (D), midgut replication (E), and salivary gland dissemination (F) were measured. Asterisks indicate significant differences (*p<0.05, **p<0.01). The denominator used to calculate midgut infection rates was the number of mosquitoes sampled, and the denominator for dissemination rates was the number of midguts with detectable virus titre. Denominators are shown (n).

**Figure 4:**
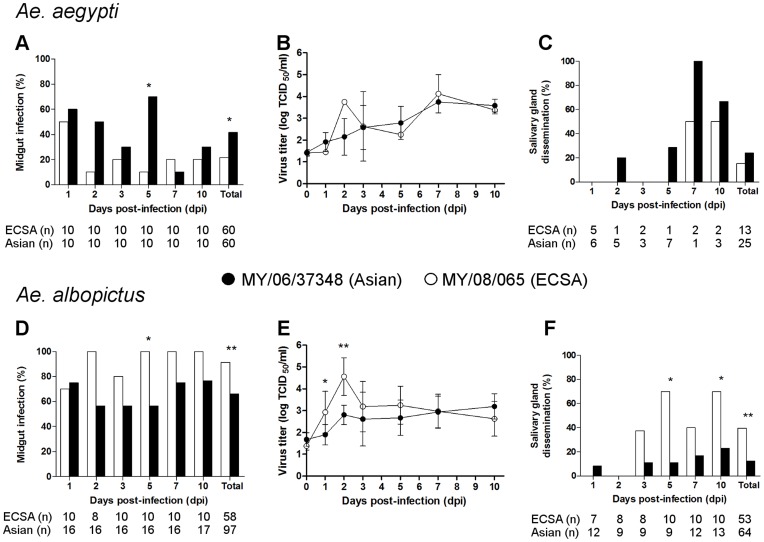
Comparative infection, replication kinetics, and dissemination of Malaysian CHIKV strains in either *Ae. aegypti* or *Ae. albopictus*. The CHIKV isolates MY/06/37348 (Asian, • and black bars) and MY/08/065 (ECSA, ○ and white bars) were used. Using *Ae. aegypti*, infection rates of midguts (A), replication in midguts as measured by a TCID_50_ titration assay with plotted means ± SD of triplicates (B), and dissemination rates in salivary glands (C) were determined. Following infection of *Ae. albopictus*, midgut infection (D), midgut replication (E), and salivary gland dissemination (F) were measured. Asterisks indicate significant differences (*p<0.05, **p<0.01). The denominator used to calculate midgut infection rates was the number of mosquitoes sampled, and the denominator for dissemination rates was the number of midguts with detectable virus titre. Denominators are shown (n).

## Results

### Genotypic Characterisation of Malaysian CHIKV

The full coding sequences (11,172 nt) of the isolates from Bagan Panchor, MY/06/37348 and MY/06/37350 (Asian genotype), and the isolates from Johor, MY/08/065 and MY/08/068 (ECSA genotype), were compared (Table 1). Within each genotype, MY/06/37348 and MY/06/37350 had 99.97% amino acid similarity (1 amino acid difference over the full coding sequence), and MY/08/065 and MY/08/068 had 99.92% similarity (3 amino acid differences). Overall, there was 93.7% nucleotide and 96.8% amino acid similarity between the two genotypes, with the highest number of amino acid differences seen in nsP3 (6.6%), 6 K (6.5%), E3 (6.3%), and E2 (4.3%). A notable difference was the presence in the Asian strains of a 21 nucleotide deletion in nsP3, leading to a 7 amino acid deletion at positions 376–382. However, sequences from four other isolates from the same Bagan Panchor outbreak, deposited by another institution in GenBank (accession numbers EU703759-62), do not have the deletion. To exclude the possibility of the deletion arising from laboratory passaging, amplification and sequencing was performed directly from the original patient serum from which MY/06/37348 and MY/06/37350 were isolated, as well as two other culture-positive serum samples from the same outbreak, MY/06/37337 and MY/06/37352. The nsP3 deletion was present in all four serum samples, confirming that the deletion was present in the outbreak virus strain.

### Phylogenetic Analysis

The phylogenetic tree showed the three main genotypes of CHIKV, West African, ECSA, and Asian (Figure 1). The Bagan Panchor strains MY/06/37348 and MY/06/37350 grouped in the Asian genotype. The epidemic ECSA strains from 2005–2010 were further divided into the Indian Ocean and Indian sublineages [21]. The ECSA strains MY/08/065 and MY/08/068 were within the Indian sublineage, and clustered with strains from Kerala (India), Taiwan, Thailand, China, and Singapore.

### Comparative Replication Kinetics of Malaysian CHIKV Strains in Cells

The virus titres of MY/06/37348 and MY/08/065 in Vero, RD, C6/36 and CCL-125 cells were quantified. Both viruses replicated equally well in Vero cells, reaching a peak titre of 6.5–6.8 log_10_ TCID_50_/ml at 40–48 hpi (Figure 2A). In RD cells, both viruses reached a similar peak titre of about 6.8 log_10_ TCID_50_/ml at a similar rate by 48 hours, before declining (Figure 2B).

In C6/36 (*Ae. albopictus*) cells, there were significant differences in peak titres of the two viruses (Figure 2C). MY/06/37348 reached a peak titre of 7.2 log_10_ TCID_50_/ml at 48 hpi, before declining. MY/08/065 attained a higher peak of 8.1 log_10_ TCID_50_/ml at 64 hpi, and maintained titres which were significantly greater by 1.2–1.5 log_10_ TCID_50_/ml up to 96 hpi. We were unable to infect CCL-125 (*Ae. aegypti*) cells with CHIKV at an MOI of 0.1. Using an MOI of 1, there was limited virus titre during early infection and short-lived replication, with peak titres of 3.7–4.4 log_10_ TCID_50_/ml less than those achieved in C6/36 (Figure 2D). In CCL-125, the peak titre of 3.5 log_10_ TCID_50_/ml for MY/06/37348 was achieved at 24 hpi, 16 hours earlier than the 3.7 log_10_ TCID_50_/ml maximum for MY/08/065. There were no significant differences between the peak levels attained. Virus titres steadily declined to below starting levels by 48 hpi and 64 hpi for MY/06/37348 and MY/08/065, respectively.

Overall, the mammalian Vero and RD cells were highly and equally permissive to both Malaysian CHIKV strains. In C6/36 cells, the ECSA strain MY/08/065 reached and maintained significantly higher titres than the Asian strain MY/06/37348. Both viruses replicated equally poorly in CCL-125 cells.

### Comparative Replication Kinetics of Malaysian CHIKV Strains in Mosquitoes

*Ae. aegypti* and *Ae. albopictus* mosquitoes were infected with either MY/06/37348 or MY/08/065. Virus titres were determined from culture of midguts (to demonstrate infection) and salivary glands (to show dissemination) at defined time-points. At each time-point after infection, 10–17 mosquitoes were sampled (apart from one time-point, where n = 8).

The replication of each virus in different mosquito species was compared (Figure 3). The MY/06/37348 Asian strain infected *Ae. albopictus* at higher overall rates (64/97, 66.0%) than *Ae. aegypti* (25/60, 41.7%; p = 0.005), predominantly at later stages of infection, at 7 and 10 dpi (Figure 3A). There were no significant differences in midgut titre (Figure 3B) or total salivary gland dissemination rates (12.5% vs 24.0%, p = 0.21) (Figure 3C). The MY/08/065 ECSA strain also infected *Ae. albopictus* (53/58, 91.4%) at higher rates than *Ae. aegypti* (13/60, 21.7%; p<0.001), overall and at all time-points but 1 dpi (Figure 3D). While some of the virus found in the midgut in the early days is from the blood meal, MY/08/065 replicated to higher titres earlier in *Ae. albopictus*, significantly so at 1 dpi (Figure 3E), and there was a trend to greater dissemination in *Ae. albopictus* (21/53, 39.6%) than *Ae. aegypti* (2/13, 15.4%; p = 0.12) (Figure 3F). Therefore, both CHIKV strains showed higher infection rates in *Ae. albopictus* than *Ae. aegypti*.

The replication of both viruses was then compared within each mosquito species (Figure 4). In *Ae. aegypti*, total infection by MY/06/37348 was greater than MY/08/065 (41.7% vs 21.7%, p = 0.03) (Figure 4A), although midgut titres were similar (Figure 4B). Salivary gland dissemination rates were also similar (15.4% vs 24.0%, p = 0.69) (Figure 4C). In *Ae. albopictus*, MY/08/065 clearly infected at higher rates (91.4% vs 66.0%, p<0.001) (Figure 4D), replicated more quickly over the first 2 dpi to reach titres greater by 1.0–1.8 log_10_ TCID_50_/ml (Figure 4E), and disseminated at higher rates (39.6% vs 12.5%, p<0.001) than MY/06/37348 (Figure 4F). Therefore, the MY/08/065 ECSA strain infected, replicated, and disseminated at higher rates than the MY/06/37348 Asian strain in *Ae. albopictus*, while MY/06/37348 infected *Ae. aegypti* at a marginally higher rate than MY/08/065.

## Discussion

In Asia, where both Asian and ECSA strains now circulate, differences in replication in humans, monkeys, or mosquitoes may impact the predominance of one CHIKV genotype over another. In this study, both Malaysian Asian (MY/06/37348) and ECSA (MY/08/065) strains replicated equally well in the mammalian cell lines Vero and RD. The ECSA strain replicated to significantly higher titres than the Asian strain in *Ae. albopictus* (C6/36) cells. Both strains replicated poorly in the *Ae. aegypti* cell line CCL-125, reaching similar titres albeit at different times. Poor replication in CCL-125 was also seen in a recent study of ECSA strains [22], consistent with the original descriptions of these mosquito cell lines [23].

To confirm the *in vitro* findings, we infected Malaysian *Aedes* mosquitoes with Malaysian CHIKV strains. This is more likely to reflect natural infection dynamics in a given location than using virus strains and mosquitoes from different regions, as genetic susceptibility of *Ae. albopictus* to CHIKV may vary by geography [24], [25]. We found that both CHIKV strains infect *Ae. albopictus* at a higher rate than *Ae. aegypti*, as previously shown [25], [26], and this was particularly marked with the ECSA strain. Furthermore, the ECSA strain infected, replicated, and disseminated at higher rates in *Ae. albopictus.* The Asian strain infected *Ae. aegypti* marginally better than the ECSA strain, which has not been previously shown. This supports existing field data on the likely vectors involved in the Malaysian outbreaks: *Ae. aegypti* was identified in the Bagan Panchor outbreak of Asian CHIKV [27], while the ECSA outbreaks were likely caused by *Ae. albopictus*, which predominate in the rural areas mainly involved [18], [28].

In alphaviruses, mosquito adaptation determinants map to glycoproteins E2 and E1, which mediate receptor binding and membrane fusion, respectively [29]. There were differences between Malaysian Asian and ECSA strains at potential mosquito adaptation determinants (Table 1), in E1–98 [30], E1–226 [13], E2–118 [31], [32] and E2–207 [33]. The E1-A226V change found in ECSA strains increases infectivity and dissemination of CHIKV in *Ae. albopictus*, but has inconsistent effects in *Ae. aegypti* [13], [34]. The E1-98T residue, found only in the Asian genotype and seen in our Malaysian strains, limits the adaptive effect of E1-A226V. Introduction of both E1-T98A and A226V, present in our ECSA strain, into a Malaysian Asian strain ML06 increased adaptation to *Ae. albopictus*, with no effect on *Ae. aegypti* [30]. Of note, this ML06 clone was based on a Bagan Panchor strain MY002IMR/06/BP (EU703759) without the nsP3 deletion present in our isolates. Recently, epidemic ECSA strains with E1-226A and E1-226V were shown to infect C6/36 cells similarly, and reached higher titres than the prototype ECSA Ross strain [22]. This suggests that other unidentified genetic determinants also contribute to *Ae. albopictus* adaptation in the ECSA lineage. This evolved adaptation will impact regions where *Ae. albopictus* populations are increasing [35], [36]. Where both genotypes co-exist, this may lead to displacement of Asian strains by ECSA strains.

The nsP3 protein is involved in negative strand RNA synthesis [37]. Deletions in the nsP3 hypervariable C terminus domain, which includes the deleted sites 376–382 in our Asian strains, are generally well tolerated by alphaviruses. Nevertheless, these deletions may reduce Sindbis virus infection of C7–10 (*Ae. albopictus*) cells [38]. Notably, the Indonesian CHIKV strain 0706aTW (FJ807897) from 2007, the most closely related sequence to the Malaysian Asian strains (Figure 1), had a deletion in a similar position, at codons 379–382 [8]. This suggests earlier spread of this Asian CHIKV from Indonesia to neighbouring Malaysia, with subsequent loss of a further 3 codons. Alternatively, as this deletion was absent in the few available Asian CHIKV sequences before 2006, it may be a recent evolutionary change in Asian isolates. The biological effects of this nsP3 deletion need to be determined.

Relatively little is known about CHIKV in mosquito saliva. Our data showed low dissemination rates, low salivary gland titres of 1.3–3.7 log_10_ TCID_50_/ml with no significant differences between the genotypes, and a short extrinsic incubation period of 2 days. Other studies also show low viral levels of 45±64 FFU/mL [39] and 0.5–3.3 log_10_ PFU/mL [40]. Dissemination rates in mosquito experiments are influenced by blood meal titres [26]. The blood meal titre of 4.5 log_10_ TCID_50_/ml used in our study was appropriate, as it is comparable to the median viral load of 4.7 log pfu/mL (equivalent to 4.9 log_10_ TCID_50_/ml) reported in CHIKV patients in Singapore [41]. As increased dissemination rates appear to be important in the adaptation of ECSA to *Ae. albopictus*, definitive study of inter-genotypic differences in dissemination and salivary titres are required with higher blood meal titres.

In this study, we compared replication of strains from each of the distinct Asian and ECSA CHIKV genotypes found in Malaysia. While *Ae. albopictus* was a better laboratory vector for both CHIKV genotypes than *Ae. aegypti*, the ECSA strain showed greater adaptation to *Ae. albopictus* than the Asian strain, while the Asian strain infected *Ae. aegypti* at a marginally higher rate than the ECSA strain. The genetic differences between the two genotypes include determinants of mosquito adaptation identified in other alphavirus studies. Our findings are consistent with the reported involvement of different vectors transmitting different genotypes in Malaysia, which caused human outbreaks of varying magnitude. In conclusion, transmission and epidemiology of CHIKV is critically influenced by virus strain and mosquito species. This has implications for areas with more than one circulating CHIKV genotype and varying relative proportions of different mosquito species.

## Supporting Information

Table S1 Primers used for obtaining the full coding sequence of CHIKV isolates. Previously published primers were used [5], with modifications (marked *). Nucleotide positions are based on the prototype S27 strain.
(DOCX)
