# Supplementary material for: Genotypic and Phenotypic Characterization of Chikungunya Virus of Different Genotypes from Malaysia
Source: PLoS One. 2012 Nov 27;7(11):e50476. doi: 10.1371/journal.pone.0050476 (PMC3507689; doi:10.1371/journal.pone.0050476)
Supplement: Table S1 — Primers used for obtaining the full coding sequence of CHIKV isolates. Previously published primers were used [5], with modifications (marked *). Nucleotide positions are based on the prototype S27 strain. (DOCX) [file pone.0050476.s001.docx]

**Supporting Information**

**Table S1. Primers used for obtaining the full coding sequence of CHIKV isolates.** Previously published primers were used [5], with modifications (marked *). Nucleotide positions are based on the prototype S27 strain.

| **Primer** | **Targeted region** | **Nucleotide position** | **Sequence (5’ to 3’)** | **Annealing temperature (ºC)** |
| --- | --- | --- | --- | --- |
| 1F | 5’ NTR | 18 | CACGTAGCCTACCAGTTTCTTA | 52 |
| 1R* | nsP1 | 869 | GGAACACCGATGGTAGGTG |  |
| 2F | nsP1 | 616 | AACCCCGTTCATGTACAATGC | 52 |
| 2R* | nsP1 | 1428 | ACAAAGCTGTCAAACTCGGC |  |
| 3F | nsP1 | 1317 | CACTGACCTGCTGCTGTCTATG | 52 |
| 3R | nsP2 | 2130 | AGTCCTGCAGCTTCTTCCTTC |  |
| 4F | nsP1 | 1412 | CGAGTTTGACAGCTTTGTGGTA | 52 |
| 4R | nsP2 | 2227 | ATGACTGCAATTTTGTATGGGC |  |
| 5F* | nsP2 | 1958 | TACAACGAAAGAGAGTTCG | 55 |
| 5R | nsP2 | 2709 | TCCACTACAATCGGCTTGTTG |  |
| 6F* | nsP2 | 2533 | CGGCTTCTTCAATATGATGCAG | 55 |
| 6R | nsP2 | 3343 | TCCAGGCCTATTATCCCAGTG |  |
| 7F | nsP2 | 2577 | AACATCTGCACCCAAGTGTACC | 55 |
| 7R* | nsP2 | 3542 | CTTTTACTGGGCGGTGTTCG |  |
| 8F* | nsP2 | 3324 | ACTGGGATAATAGGCCTGGAGG | 55 |
| 8R* | nsP3 | 4129 | GCACTCTTCATCGTTCTTCGC |  |
| 9F | nsP2 | 3885 | GAACGAGTCATCTGCGTATTGG | 55 |
| 9R | nsP3 | 4725 | ATATCTCTGCCATATCCACTGC |  |
| 10F | nsP3 | 4458 | TCTTTACAGCCATGGACTCGAC | 55 |
| 10R | nsP3 | 5273 | CGACAGGTACGGTGCTCATTAC |  |
| 11F* | nsP3 | 5023 | ATCGCGCGTAAGTCCAAGG | 55 |
| 11R | nsP4 | 5874 | TCTACTTTGCGCGACTGATACC |  |
| 12F | nsP3 | 5630 | ACGGACGACGAGTTACGACTAG | 55 |
| 12R* | nsP4 | 6082 | TTCCCAGTATTCTTGGTTGCATG |  |
| 13F* | nsP4 | 6360 | ATCAGAAGCGCTGTACCGTC | 50 |
| 13R | nsP4 | 6936 | AACTTGAAGCGCGTACCTGTC |  |
| 14F | nsP4 | 6732 | TCATAGCCGCACACTTTAAGC | 55 |
| 14R* | junction | 7521 | TAGGTAGCTGTAGTGCGTACC |  |
| 15F* | nsP4 | 7331 | AGATGGCAACGAACAGGGC | 55 |
| 15R* | C | 8093 | GAAGCGTCGGACTTCATGTGC |  |
| 16F | C | 7910 | TCGAAGTCAAGCACGAAGG | 55 |
| 16R | E2 | 8670 | GTCTGTCGCTTCATTTCTGATG |  |
| 17F | E3 | 8459 | TGCTTGAGGACAACGTCATGAG | 55 |
| 17R | E2 | 9240 | TTTGTGATTGGTGACCGCG |  |
| 18F | E2 | 9093 | AGTCCGGCAACGTAAAGATCAC | 55 |
| 18R | 6K | 9861 | AAAGGTTGCTGCTCGTTCCAC |  |
| 19F | E2 | 9648 | AGTTGTGTCAGTGGCCTCGTTC | 55 |
| 19R | E1 | 10403 | TAAAGGACGCGGAGCTTAGCTG |  |
| 20F | E1 | 10145 | ACAAAACCGTCATCCCGTCTC | 55 |
| 20R | E1 | 11158 | TGACTATGTGGTCCTTCGGAGG |  |
| 21F | E1 | 10959 | CAGCAAGAAAGGCAAGTGTGC | 56 |
| 21R* | 3’ NTR | 11798 | TACGTCCCTGTGGGTTCG |  |
